# Pustule Maligne

**Published:** 1855-06

**Authors:** 


Art. III.—*Pustule Maligne. An Inaugural Essay* submitted to the Trustees and Faculty of the College of Physicians and Surgeons of the University of New York, for the Degree of Doctor in Medicine. By Daniel Wadsworth Wainwright, of New York city.

This is a neat pamphlet of thirty pages. It opens with a French title, two dedications, a salutatory, and an exordium; and concludes with a couple of pages devoted to addenda. But notwithstanding this "affectation thick," Dr. Wainwright has produced a paper which bears the impress of great industry and research.

He furnishes a list of thirty authorities, and alludes to many more whose opinions he has endeavored to embody in his dissertation for the doctorate. Of course there is but little space unoccupied in which our author can show his own paces, but he is modest and natural enough after he gets under way. He has selected a subject of practical interest, and has succeeded in placing in good form much that is valuable concerning it. Apart from the Grand Overture which precedes the body of the thesis it is a very creditable production. D. W. Y.

